# COVID-19’s myths, facts, concerning and obstinate posts on social network, and the mental health status of social network users in Bangladesh

**DOI:** 10.1371/journal.pmen.0000014

**Published:** 2024-06-24

**Authors:** A. F. M. Mahmudul Islam, Khandaker Tanveer Ahmed, Md. Abu Raihan, Tania Ahmed, Md. Selim Hossain, Md. Khairul Alam Eshad, Md. Hasan Mahmud, Pabittra Kumar Shill, Shahenul Islam, Md Afjalus Siraj

**Affiliations:** 1 Department of Pharmacy, Gono Bishwabidyalay, Savar, Dhaka, Bangladesh; 2 Department of Statistics, Jahangirnagar University, Savar, Dhaka, Bangladesh; 3 Department of Microbiology, Jahangirnagar University, Savar, Dhaka, Bangladesh; 4 Vascular Biology Center, Medical College of Georgia at Augusta University, Augusta, GA, United States of America; 5 Department of Pharmacy, Dhaka International University, Satarkul, Badda, Dhaka, Bangladesh; 6 Department of Pharmaceutical Sciences, The Daniel K. Inouye College of Pharmacy, University of Hawaii at Hilo, Honolulu, HI, United States of America; University of Sousse Faculty of Medicine of Sousse: Universite de Sousse Faculte de Medecine de Sousse, TUNISIA

## Abstract

Myths, misinformation, facts like posts spread by social media during COVID-19 pandemic had an enormous effect on psychological health. This study aimed to investigate social media based COVID-19’s posts and the psychological health status of participants. A cross-sectional, online survey-based study was conducted in between April to October 2021 using a structured and semi-structured questionnaire, predominantly involving 1200 active social network users in Bangladesh. Depression, anxiety, and stress were assessed using the Depression, Anxiety, and Stress Scale (DASS-21), while the Insomnia Severity Index (ISI) measured insomnia severity for selected participants. Internal reliabilities were calculated with Cronbach’s alpha coefficients (cut-off point 0.70). Unrelated multivariate logistic regression explored correlations among outcome errors, with the model assessing the impact of selected independent variables on mental health. The findings demonstrated that 27.8% individuals spread facts whereas 7.4% spread myths and misinformation about COVID-19 on social networks. Furthermore, 28.1% and 36.7% shared obstinate and concerning posts respectively. The prevalence of depression, anxiety and stress symptoms, ranging from mild to extremely severe, were 43.9%, 30.9%, and 23.8% respectively. However, 2.8% had severe level of insomnia. Facts, myths, tour attending, and no mask group photos were significantly associated with anxiety, and less likelihood of experiencing anxiety. Interestingly, circulating such activities on social networks had no significant association with depression, stress, or insomnia. The spread of misinformation on social media undermines any efforts to contain COVID-19 infection. The findings hugely recommend of using fact checking facilities and adaptation to the pandemic situations to maintain lower prevalence of depression, anxiety, stress and insomnia.

## Introduction

The advent of the 2019 coronavirus disease (COVID-19) has not only posed an unprecedented global health crisis but has also brought about a unique set of challenges transcending the boundaries of conventional disease outbreaks. Unlike its predecessors, the rapid spread of COVID-19 has defied expectations and prompted swift and cooperative responses globally [1,2]. In addition, today’s most widely used, popular, and frequently utilized digital media platforms for regular personal connection and communication are Facebook, Instagram, Twitter, YouTube, and WhatsApp. These social media platforms have profoundly transformed interpersonal relationships by enabling users to engage and disseminate information, viewpoints [3]. Social media became the primary source for gathering and disseminating information during the pandemic since individuals relied solely on it as a communication tool [4]. However, social media platforms are infamous for disseminating misinformation and myths [5] Myths about health and illness are typically created by conspiracy theories, fictitious stories, and unproven scientific claims that circulate on social media and eventually mislead people [6]. In response to the rapid spread of false information about the COVID-19 virus, which eventually led to psychological problems in people [7], the World Health Organization (WHO) declared during the COVID-19 pandemic that it had to combat both the infodemic on social media and the COVID-19 virus. This presented a major challenge, considering that the general public places three times more faith in fabricated and misinformation shared on social media platforms than in any information that is true. Consequently, these platforms propagate falsehoods pertaining to health and diseases [8]. During disease epidemics such as Ebola, Dengue fever, H1N1 flu, and Zika, news media became a crucial source of information [9–12]. As billions are restricted or isolated within their homes to combat the condition, "online screen engagement" exposure has increased in tandem with media saturation [13]. Social media networks are notorious for spreading misinformation and myths [5]. During the COVID-19 epidemic, individuals were observed with their eyes confined on watching television, computer systems, or mobile screens while capturing coronavirus-related news feeds. This was frequently seen on a "compulsive" tone, including the desire to stay up to date on every element of the condition, which exacerbates psychological discomfort and bodily uncomfortable [14].

Despite concerted efforts to contain the virus, the pandemic has proven relentless, impacting human lives on an unprecedented scale. The resultant measures, such as social distancing and restrictions on daily activities, have not only disrupted the normal course of life but have also cast a profound shadow on mental health across diverse populations. As individuals grapple with feelings of insecurity and unhappiness during this tumultuous period, the common response of seeking close interactions becomes more pronounced [15]. However, the imposed solitude, necessitated by social distancing measures, introduces a new layer of anxiety, leading to concerns about loneliness and uncertainty [16,17]. This initiative not only had a negative impact on all ongoing activities, but it also had a catastrophic impact on people’s mental health. An individual’s life has been drastically changed as a result of social distancing and the discontinuation of important normal activities [16]. One further significant global public health concern during this epidemic is the rise in mental health issues in every civilization and age group worldwide [18–25]. Since the start of the epidemic, epidemiological statistics in Bangladesh have showed that mental health concerns are prevalent as a result of the COVID-19 pandemic associated social isolation [26,27]. Fear, which was identified early in the epidemic, was one of the primary elements that contributed to these mental health implications for Bangladeshis [28]. According to several studies, COVID-19-related worries and fears in Bangladeshi populations are associated with greater COVID-19 anxiety, generalized anxiety, depression, and turn down mental well-being [29–32]. Apart from these psychological impacts, recent studies have shown that excessive use of social media networks had negative impact on mental health of young people and children [33,34]. Furthermore, misinformation related to COVID-19 spread through digital media especially social network, and traditional media like TV have induced anxiety, depression, and fear among its users [35–37]. Individuals who were exposed to media on a regular basis were found to have a relatively greater prevalence of mental problems [37–40]. Accordingly, one of the most important things to do during a health crisis is to spread the facts in order to lessen the impacts [41]. Apart from these psychological impacts, recent studies have shown that excessive use of social media networks had negative impact on mental health of young people and children [33,34]. Furthermore, misinformation related to COVID-19 spread through digital media especially social network, and traditional media like TV have induced anxiety, depression, and fear among its users [35–37]. Individuals who were exposed to media on a regular basis were found to have a relatively greater prevalence of mental problems [37–40]. Accordingly, one of the most important things to do during a health crisis is to spread the facts in order to lessen the impacts [41].

In a very uncertain situation with gradually increasing COVID-19 death toll incidence, this study aimed to unravel the prevalence of social media-based facts, myths and misinformation during the COVID-19 pandemic in Bangladesh, with a keen focus on understanding the psychological health status of individuals actively engaging in such online content dissemination. In doing so, the research aspires to contribute valuable insights that extend beyond the immediate context, informing strategies for mental health support and public communication during times of global health crises.

### Literature review

Heightened insecurity and unhappiness during periods of uncertainty often prompt individuals to seek close interactions, and imposed solitude, as witnessed during the COVID-19 pandemic, may intensify anxiety, leading to mental health disorders [15]. The pandemic-induced disruptions, encompassing social distancing and curtailed activities, had profound negative consequences on mental health [16,17]. Factors contributing to escalating psychological distress included fears of contracting the virus, limited access to treatment, high virus-related mortality rates, and uncertainty regarding control and vaccine availability. Additionally, challenges such as social event cancellations, financial losses, disrupted routines, and incessant exposure to news exacerbated the situation, leading to severe mental health consequences, including instances of suicide. Globally, a surge in mental health issues has been a significant concern during the pandemic, evident across civilizations and age groups. In Bangladesh, epidemiological data underscore the prevalence of mental health concerns linked to COVID-19-related social isolation [26,27]. Fear emerged as a pivotal element contributing to mental health implications for Bangladeshis [28], with studies establishing associations between COVID-19-related worries and heightened anxiety, depression, and reduced mental well-being [29–32]. Tertiary-level students, undergoing a critical transitional phase, are particularly vulnerable to psychological impacts [42–44], with studies indicating elevated levels of anxiety and depression [45].

Social stigma surrounding COVID-19, fueled by hoaxes and rumors, further hindered containment efforts [46] with instances of non-compliance with quarantine measures and risky behaviors emerged [47], driven by misguided remedies and unfounded beliefs [48]. In combination with food and vitamins to increase immunity, several studies concentrated on so-called remedies such as magical mineral solutions, which entailed mixing the sodium chlorite solution in citric acid [49,50] or using bleach or alcohol to boost immunity and heals [51]. Other recorded remedy stories included drinking tea with cow urine or manure in India [52], camel urine mixed the lime in Saudi Arabia, as well as therapeutic plants in Africa [53].

Most of the former studies [54–57] demonstrated the prevalence of disease and health related misinformation on different social media networks. Previous findings showed that misinformation had the tendency to avoid television, newspaper like established and traditional media while spreading. However, in most cases information was acquired mainly through digital media, including the internet and social media [58–60]. The digital social networks rather than the newspaper, radio, and television like traditional media systems, has been considered a malicious medium for spreading mis- and disinformation [58,61,62].

Several studies in recent years on COVID-19 have explained the negative impacts of COVID-19’s myths on psychological health of individuals. A study [63] found a positive association between belief in COVID-19’s misinformation on digital platforms and higher level of depression. Social media has a detrimental effect on mental health and psychological well-being, according to a study [64]. Swami et al. also established a positive association between beliefs in conspiracy theories and stress, anxiety [65]. A study by Mongkhon et al. reported that people who were exposed to COVID-19-related information for 3 or more hours per day were more likely associated with developing anxiety, depression and insomnia [66].

Since the topic of current research study is circulating COVID-19’s myths, facts, concerning and obstinate posts on social network, it is predictable that people sharing such posts on their social networks will have distinctive psychological health status during the pandemic of COVID-19. Furthermore, we also expect that exposure to social networks on daily basis in the pandemic situation will be associated with sharing facts, myths, concerning and obstinate posts related to COVID-19.

## Methods

### Study design and procedure

The present study used a cross-sectional and online survey-based study using a structured and semi structured questionnaire between April to October 2021. The study targeted active social network users typically on social network. Participants were selected and observed by the recruited and trained group members having coordinators who directly informed every progress to the person who designed this study. Before conducting this study, the participants were selected from social network friends list of each of group members. Those persons on social networks were selected from their posts related to the corona virus or measures, suspected posts of myths and misinformation or facts, memes or trolls regarding COVID-19, concerning posts on COVID-19’s scenarios. The study conductors together with the group coordinators also selected participants of this study who attended tours, ceremonies, anniversaries, family programs, get together programs with their friends and snapped group photos without wearing masks or maintaining social distances.

### Inclusion & exclusion criteria

The eligibility criteria included (i) active social network users (ii) Bangladeshi origin (ii) shared different posts and/or activities related to COVID-19 on social networks during COVID-19 pandemic; and exclusion criteria are like initially consented but submitted response without answering all the study related questionnaires, or submitted response immediately after commencing the questionnaire answering session.

### Sampling procedure

Raosoft Sample Size Calculator [67] was used for determining sample size, where margin of error was 5%, confidence interval was 95%, population was 4,50,00,000 (social network users by 2021) [68] and expected response was 50%. 385 was the minimum sample size and a sample of 1200 active social network users were included in this study (three times of estimation) to enhance the statistical significance.

### Questionnaire administration

After completion of the initial targeted selection procedures, the link of the google online study questionnaire was shared by the study conductors via different social network platforms like Facebook, WhatsApp, Instagram etc. to acquire sufficient response from online self-responding circumstances from April 6, 2021 to August 6, 2021. A total of 1809 participants initially agreed to participate in this study, voluntarily without any provocations for incentives. After excluding 609 incompletely or partially submitted responses from the online section, only 1200 were included in the final study analysis. Next to the successful completion of online survey study sections, we also concomitantly observed the publicly shared posts related to facts, myths, and hoax messages of COVID-19, publicly posted trolls and memes regarding COVID-19, concerning posts related to COVID-19 upon taking consent on the consent statement of each participant.

### Definitions of different study related terms

Health myths may be defined as any information regarding health beefed up by embellishing too many false or misinformation and thus widespread as appealing intuitive on a suitable media but are not buttressed by any available scientific evidence [69–71]. According to Wardle and his colleagues (2021), the occurrence of anyone’s fake or incorrect information sharing by unintentionally and unwittingly is called misinformation [72]. As long as myths disseminate, it will bring and spread misinformation. We identified different posts regarding COVID-19 misinformation that were shared and spread by the social network users we studied and these posts have no strong scientific evidence. Therefore, we have denoted those posts by the term called myths and misinformation. True information about anything may be referred to as facts [73]. Science oriented facts can sometimes be verified by scientific evidence available. Facts elucidate details about real occurrences and situations. In this study, we considered facts as only true verified information of COVID-19. Recreational activities referred to the activities people do to freshen their bodies and minds and make their leisure time more fascinating and amusing [74]. According to our study, recreational activities are comprised of posts of tours’ check-in, attending ceremonies and get-together programs, no-mask group photos, memes and trolls sharing related to COVID-19 etc. During the lockdown period of second wave of COVID-19 pandemic in Bangladesh, people passed most of their leisure time by scrolling Facebook and posted different recreational activities indicating posts encircling COVID-19 for their amusements. Obstinacy is individual’s self-willed characteristic towards the pursuance of a desired goal [75]. Obstinacy also tends to eliminate any restraining force. In our study we considered these recreational activities related posts as obstinacy of social network users in this pandemic lockdown situation. Social network users having obstinacy inclined not to obey any preventive measures of COVID-19.

### Content analysis & identification of facts, myths and misinformation

To identify whether the contents shared or wrote on social network platforms by the participants are facts, myths and misinformation of COVID-19, we took help from the searches of the web to find content named BuzzSumo [76]. All the researchers decided to use this content-based web search to verify the shared or posted contents of participants and since BuzzSumo is user-friendly, it helped make this study more transparent, reproducible, and free of charges search was possible for such investigation. Giving input of some content keywords of the suspected social networks’ posts on BuzzSumo ultimately identified the facts, myths and misinformation [76]. The veracity of COVID-19 information and myths found in different posts shared by participants was also justified by comparing with updated contents of MythBusters section of the website of WHO which can be easily accessed by anyone [77].

Each post shared on social networks was diligently scrutinized and reviewed by two competent researchers (both were pharmacists) who were unknown to the users who shared COVID-19 related posts on social networks. This process was devised from two previous studies [78,79]. Shared posts were appointed in four categories according to the content, and scientific evidences⁠. The authors have conducted a thorough literature review, cross-referencing to identify if the information shared on social networks was in line with scientific journals and authoritative institutions such as the Ministry of Health of Bangladesh, the Institute of Epidemiology, Disease Control and Research (IEDCR), FDA, CDC, and WHO. This process ultimately helped in distinguishing COVID-19’s facts from its myths.

Therefore, in this study we analyzed and appointed the shared posts on social networks in four pre-established and well-defined categories:

1) COVID-19’s myths: fallacious, irrelevant and fabricated information about COVID-19 that were noticeably different from those that were conforming to scientific standards. Specifically, any shared posts on social network spreading information that did not replicate a scientific fact or partially replicate scientifically validated information that might have caused potential misunderstandings, was categorized as myths.

2) COVID-19’s facts: any posts indicating general truth and scientific evidence-based information about COVID-19, was categorized as facts.

3) Obstinate posts: posts indicating tour or ceremony or get together attend, no mask group photos and sharing memes and trolls of COVID-19.

4) Concerning posts: posts indicating individuals’ concern about COVID-19 situation during the second wave of COVID-19.

### Questionnaire design

In order to clarify ambiguity of open ended, Likert scale and multiple-choice questions, a pilot study was performed among 60 participants before commencing the data collection process of this study. Some questions were modified in response to the feedback of participants. The data that we obtained from pilot study, was not included in the results of this research. The final questionnaire we designed has actually three separate parts. The first part mainly focused on collecting socio-demographic data including gender, age, marital status, educational qualification, present address, and occupation. The second part of the study questionnaire has different personal life preference questions during this COVID-19 pandemic situation. Personal life preference related activities were collected by asking questions concerning the total number of tours he or she may go during the COVID-19 pandemic, total numbers of attended ceremonies/ anniversaries/ family programs during this pandemic, total numbers of attended get-together programs with friends or other members during this pandemic situation. The third part has two validated and reliable psychometric indices, one of which is DASS-21 to assess depression, anxiety, and stress, and another scale named Insomnia Severity Index (ISI) for measuring the nature, and severity of insomnia among some selected participants. In order to increase consistency, participants’ understating, and better responses, the whole questionnaire was translated in Bengali format (S1 File). After the completion of the study, the study questionnaire was translated back into English (S2 File).

### Ethics and participant’s consent

This research was authorized by the ethical review board of the Gono Bishwabidyalay, Savar, Dhaka, Bangladesh [Reference Number: CMR/EC/003]. Prior to enrollment in this study, informed consent was obtained from each individual who met the inclusion criteria. All data were obtained anonymously, and the privacy and confidentiality of all participant information was rigorously protected. After opening the online link of study questionnaire, participants had to read a detailed information emphasizing on the objectives of the study and the maintenance of privacy and confidentiality of the information of the participants of this study. Participants of this study complied voluntarily by providing ‘Yes’ and refused by ‘No’ on the informed consent form. Informed consent forms were automatically recorded with google Forms. All the participants of this study had to provide their consent first, after being agreed with this session they could finally access questionnaire session.

We designed a consent statement stating the specific purposes, observation ways, rationale of this study in pandemic situation in Bengali translated form. The research work was designed to carry out online self-responding circumstances. Before commencing their response to the questionnaire, we designed, every individual thoroughly reviewed the consent statement we provided them and expressed their consent by completing the answer of the following question first: *“This study will not disclose your name*, *address*, *Facebook ID*, *Instagram ID to anyone after completing the research work*. *Now are you willingly ready to provide answers to help make this study successful in this pandemic situation*? *You will remain as an anonymous person and we will only observe your publicly shared activities on your social networks for collecting some more information regarding COVID-19*.*”*

### Measures

#### Insomnia Severity Index (ISI)

Participants of this study completed the ISI, a brief self-reported seven items instrument with a five-point Likert scale for measuring the participant’s ability to understand his or her insomnia level. Its corresponding contents also act as insomnia’s diagnostic criteria of which the first three items target the assessment of participants’ symptoms of insomnia at early, middle, late stages, and the higher the scores the greater will be the insomnia severity. However, the remaining four items measure the consequences of insomnia such as current sleep pattern’s satisfaction/dissatisfaction and noticeability of sleep disturbance, sleep distressed, and interference of sleep problem with daily functioning respectively. Five-point Likert scores ranging from 0 to 4, of which 0 represents “very satisfactory sleeping pattern” or “no noticeable/distressful/interfering sleep pattern”. However, scores of 4 represent “very dissatisfactory sleep pattern” or “very much noticeable/distressful/interfering sleep pattern”. Bastien CH and his colleagues in 2001 buttressed and established that the ISI has adequate internal consistency, validity, sensitivity and reliability as a self-report measure to quantify perceived insomnia severity [80].

#### Depression, anxiety, and stress scale (DASS21)

The assessment of the psychometric characteristics was performed using the 21-item version of the Depression, Anxiety, and Stress Scales (DASS-21) among different selected social network user participants. The DASS-21 was chosen because of its reliability, good to excellent internal consistency, convergent and divergent validity, easily administrable, and ideal for both clinical and research purposes [81–83]. DASS21 scale is consisting of total three sub-scales having seven Depression items (DASS21-D), seven Anxiety items (DASS21-A), and seven Stress items (DASS21-S). Each of these 21 items has a statement and four-point Likert scale to reflect severity and scores ranging from 0 (Did not comply with me) to 3 (comply with me very much or always comply with me). Sum scores of each subscale of DASS21 are calculated by adding the scores per seven depression items, seven anxiety items, and seven stress items respectively. In order to have similar scores like DASS42, the total sum score of each subscale is multiplied by two [84].

#### Independent variables

As independent variables, standardized age, education level, marital status, occupation and smoking habit were inserted. Several COVID-19 related variables were added as independent variables, such as facts: facts and truths related to COVID-19 spread through social media by the respondent, myths: myths and false information related to COVID-19 spread through social media by the respondent, went to tour: respondent went to tour during this pandemic, attended ceremony: respondent attended any ceremony during this pandemic, get together: respondent attended any get together program during this pandemic, mask less group photo: respondent taken and posted any group photo in social media without mask during this pandemic, meme and trolls: respondent posted or shared any COVID-19 related meme or troll in social media, COVID-19 concerning posts: respondent posted any post in social media concerning COVID-19.

### Statistical analysis

Frequency analysis of the socio-demographic variables, mental health variables and COVID-19 related variables were presented to ascertain the outcomes of these variables from the collected data. Pearson product moment correlations and Pearson Chi square test for two level categorical variables were used to determine the bivariate relations between the mental health variables such as depression, stress, anxiety and insomnia, respondent’s socio-demographic characteristics and COVID-19-related variables. To measure the internal reliabilities of questions to calculate depression, stress, anxiety and insomnia, Cronbach’s alpha coefficients were calculated with cut-off point set at 0.70. Seemingly unrelated multivariate logistic regression was performed which assumes correlation among the outcome errors and so jointly models the outcomes with a system of equations. The regression model was fitted to inspect the impact of the selected independent variables on the mental health variables. In the implementation of a seemingly unrelated multivariate logistic regression to evaluate the influence of selected independent variables on mental health, potential confounding variables were systematically addressed. Age, marital status, education level, occupation, and smoking habit were identified as critical confounders, as their uncontrolled effects could distort interpretations by intersecting or overshadowing the impact of the selected independent variables. Rigorous control for these confounding factors is imperative for an accurate and nuanced comprehension of the relationship between the designated predictors and mental health outcomes within the multivariate logistic regression model. Analysis was performed using RStudio version 1.2.5001. Seemingly unrelated logistic regression was done by using ‘Systemfit’ package.

## Results

**Table 1** contains frequency analysis of the socio-demographic characteristics, COVID-19 related variables and mental health variables of the collected data where 1200 individuals were included. Mean age of the participants is 24.18 (SD = 4.96) and 93.8% were under 30 years old. Most of the participants (75.33%) were male, (68.7%) graduates, students (73.8%), married (83.5%) and non-smokers (79.8%). Of the 1200 respondents, 27.8% had spread facts related to COVID-19, 7.4% spread myths and misinformation. During this pandemic situation, 16.8% of the participants of this study went to tour, 7.4% attended different ceremonies, 8.3% had get together, 26.4% took group photos without masks, 21.7% posted or shared COVID-19 related memes and trolls, 36.7% posted or shared COVID-19 concerning posts. *Internal reliability was high above the Cronbach’s alpha coefficient cut-off point for stress*, *anxiety*, *depression*, *and insomnia*. *Stress had an internal consistency of 0*.*843*, *anxiety had 0*.*868*, *depression had 0*.*831*, *and insomnia had an internal consistency of 0*.*894*. Among the participants, 3.8% were severely stressed and 1.1% were extremely severely stressed, 5.0% were severely depressed and 4.0% were extremely severely depressed, 4.5% were suffering from severe anxiety and 5.2% were suffering from severe anxiety, suffering from severe anxiety, 8.2% had clinical insomnia (moderate severity) and 2.8% had clinical insomnia (severe). **Table 1** presents the frequency analysis performed.

**Table 1:** Demographic information, COVID-19 related activity and mental health condition.

	Frequency	Percentage
**Age**		**(N = 1200)**
Young	1126	93.8
Old	74	6.2
**Gender**		
Male	904	75.33
Female	296	24.67
**Education**		
Lower education	93	7.8
Undergraduate	275	22.9
Graduate	824	68.7
Postgraduate	7	0.6
**Occupation**		
Student	885	73.8
Job	265	22.1
Business	50	4.2
**Marital status**		
Married	1002	83.5
Unmarried	198	16.5
**Smoking habit**		
Smoker	242	20.2
Non-smoker	958	79.8
**Facts spread**	333	27.8
**Myths and misinformation spread**	89	7.4
**Tours during pandemic**	202	16.8
**Attended ceremonies during pandemic**	89	7.4
**Get together during pandemic**	100	8.3
**Group photo without mask**	317	26.4
**COVID-19-related meme/troll**	260	21.7
**COVID-19 concerning post**	440	36.7
**Stress condition**		
Normal	914	76.2
Mild	148	12.3
Moderate	79	6.6
Severe	46	3.8
Extremely Severe	13	1.1
**Depression condition**		
Normal	673	56.1
Mild	203	16.9
Moderate	216	18.0
Severe	60	5.0
Extremely Severe	48	4.0
**Anxiety condition**		
Normal	829	69.1
Mild	161	13.4
Moderate	94	7.8
Severe	54	4.5
Extremely Severe	62	5.2
**Insomnia condition**		
No clinically significant insomnia	839	69.9
Subthreshold insomnia	229	19.1
Clinical insomnia (moderate severity)	98	8.2
Clinical insomnia (severe)	34	2.8

Now a days, social media plays a vital role in spreading information rapidly to many people and hence, facts along with false information can also be circulated. As this COVID-19 pandemic situation is an unprecedented event for everyone in this world, people got panicked at the advent of it. So, social media have seen flooding of news and information being uploaded and posted in of which many are facts and truths. But unfortunately, some myths, misinformation and lies have been spread through social media by some people. This study found some kinds of facts and myths related to COVID-19 that people have shared and posted in social media. **Table 2** contains the mostly spread facts and myths by the 1200 participant’s social media account. Most of the participants posted and shared facts about preventive measures against corona virus, facts about vaccines development, whereas misinformation and myths have been spread by people mostly about false about corona virus spreading, how corona virus originated and treatment of COVID-19.

**Table 2:** Mostly spread facts and myths in social media.

	Frequency	Percentage
**Facts**		**(N = 333)**
Preventive measures against corona virus	105	31.5
Facts and updates about COVID-19 vaccines	28	8.4
Truth about corona virus origin	17	5.2
Reality of pandemic situation in Bangladesh	16	4.8
True symptoms of COVID-19	15	4.5
Updates regarding educational institutions opening	15	4.5
**Myths and misinformation**		**(N = 89)**
Wrong information about corona virus spreading	18	20.2
False information about corona virus origin	15	16.9
Misinformation about treatment of COVID-19	14	15.7
Alcohol kills corona virus	9	10.1
Misinformation about vaccines	7	7.9

**Table 3** shows a category-by-category comparison of spreading facts, myths, and mental health, along with their corresponding frequencies and percentages, where respondents were classified by gender and COVID-19 concern. Those who went on tours during the pandemic attended ceremonies during the pandemic, attended any get-togethers during the pandemic, took group photos without masks, shared COVID-19-related memes/trolls, all or any of these activities, were labeled as ’careless,’ while those who didn’t were labeled as ’careful.’ Females had slightly higher rates of sharing facts and misconceptions than males, as can be observed. In addition, females exhibited greater rates of moderate, severe, and extremely severe stress, depression, anxiety, and insomnia than males.

**Table 3:** Category-wise comparison of spreading facts, myths, and mental health.

	Male		Female		Obstinate people		Concerned people	
	Frequency	Percentage (N = 904)	Frequency	Percentage (N = 296)	Frequency	Percentage (N = 649)	Frequency	Percentage (N = 551)
**Facts spread**	239	26.40	94	31.80	94	14.5	239	43.4
**Myths and misinformation spread**	70	7.70	19	6.40	27	4.2	62	11.3
**Both facts and myths & misinformation**	16	1.77	2	0.68	8	1.23	10	1.81
**Stress condition**								
Normal	717	79.30	197	66.60	494	76.10	420	76.20
Mild	100	11.10	48	16.20	70	10.80	78	14.20
Moderate	51	5.60	28	9.50	53	8.20	26	4.70
Severe	27	3.00	19	6.40	26	4.00	20	3.60
Extremely Severe	9	1.00	4	1.40	6	0.90	7	1.30
**Depression condition**								
Normal	522	57.70	151	51.00	371	57.20	302	54.80
Mild	157	17.40	46	15.50	94	14.50	109	19.80
Moderate	154	17.00	62	20.90	117	18.00	99	18.00
Severe	45	5.00	15	5.10	36	5.50	24	4.40
Extremely Severe	26	2.90	22	7.40	31	4.80	17	3.10
**Anxiety condition**								
Normal	644	71.20	185	62.50	467	72.00	362	65.70
Mild	115	12.70	46	15.50	83	12.80	78	14.20
Moderate	71	7.90	23	7.80	44	6.80	50	9.10
Severe	30	3.30	24	8.10	21	3.20	33	6.00
Extremely Severe	44	4.90	18	6.10	34	5.20	28	5.10
**Insomnia condition**								
No clinically significant insomnia	655	72.50	184	62.20	456	70.30	383	69.50
Subthreshold insomnia	166	18.40	63	21.30	119	18.30	110	20.00
Clinical insomnia (moderate severity)	62	6.90	36	12.20	55	8.50	43	7.80
Clinical insomnia (severe)	21	2.30	13	4.40	19	2.90	15	2.70

It can be shown that careful people had slightly higher percentages of sharing facts than careless persons. Surprisingly, careful people had slightly higher rates of propagating myths than careless persons. Careless persons exhibited somewhat larger percentages of moderate and severe stress than careful ones, whereas extremely severe stress had the opposite situation. Careless people exhibited marginally larger percentages of severe and extremely severe depression than careful ones, but they both had the same percentage of moderate depression. Careful persons showed slightly greater percentages of moderate and severe anxiety than careless people, however, extremely severe anxiety had the opposite situation. Careless people experienced modestly greater percentages of moderately severe and severe insomnia than careful people, as it is observed.

**Fig 1:**
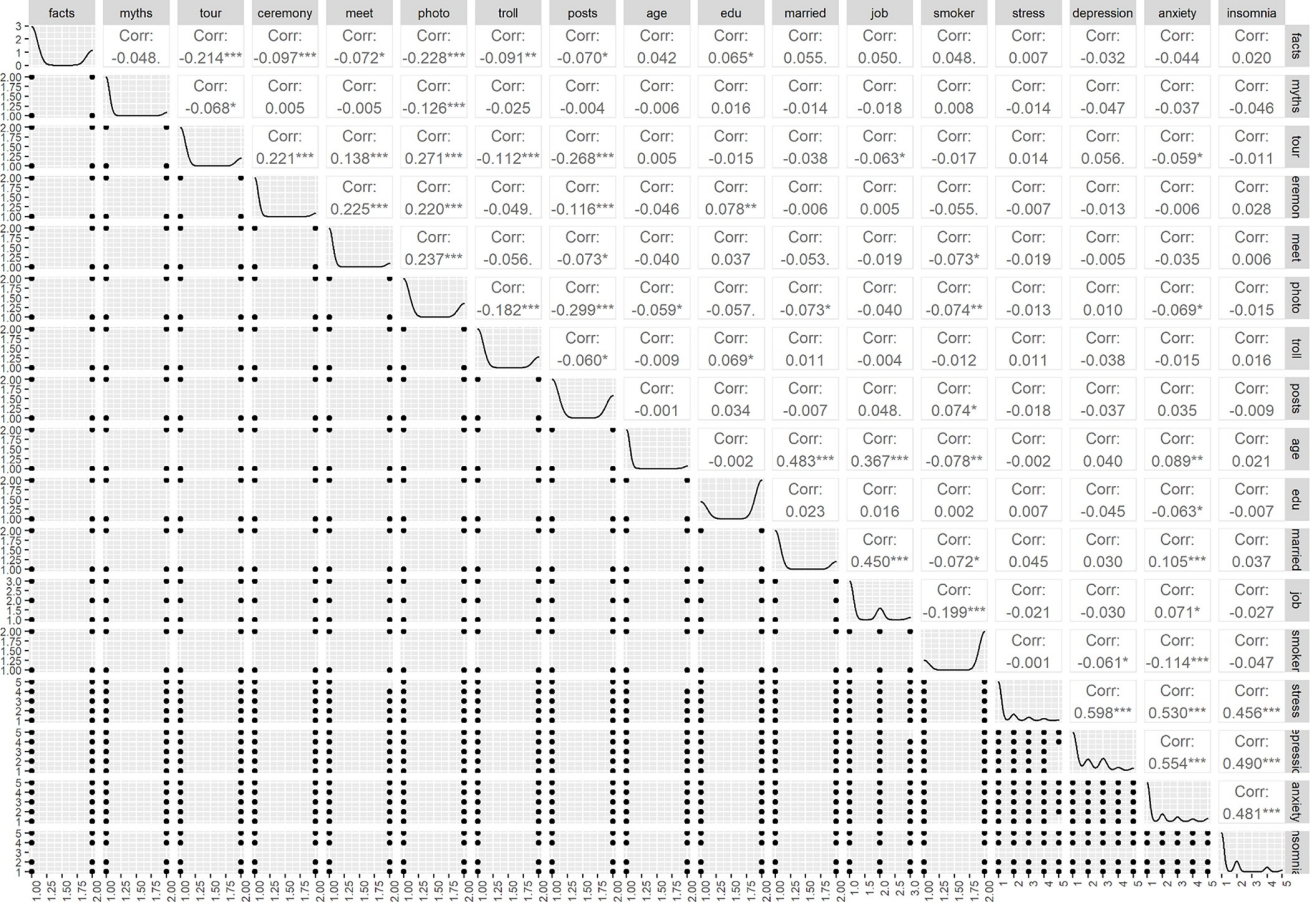
Correlation between study variables. This figure illustrates the pairwise correlation coefficients between various study variables. The density of the dots reflects the strength of the correlation, with darker shades denoting stronger relationships. Diagonal cells represent perfect correlations, marked by solid black squares.

**Table 4** presents correlations between the study variables where it is found as expectation that stress, depression, anxiety and insomnia, all are positively correlated with each other. Facts spread has negative correlation with other COVID-19-related variables, the variables indicating violation of COVID-19 preventive measures, expectedly. COVID-19-related variables as well as age, education, occupation and smoking habit are mostly negatively correlated with the mental health variables but marital status is positively correlated with these variables. **Fig 1** graphically presents the correlations between the variables and the data types of the variables. The correlation plot visually demonstrates the correlations between the study variables as presented in **Table 4**, with the upper diagonal triangle showing the correlations between the variables, lower diagonal triangle showing the scatter plots of the data under correlated variables, and the diagonal line demonstrates how the data of correlated variables are distributed.

**Table 4:** Correlations between mental health, demographic, and COVID-19-related variable.

Variables	1	2	3	4	5	6	7	8	9	10	11	12	13	14	15	16	17
1. Facts	1																
2. Myths	-.048	1															
3. Went to tour	-.214**	-.068*	1														
4. Attended ceremony	-.097**	.005	.221**	1													
5. Get together	-.072*	-.005	.138**	.225**	1												
6. No mask group photo	-.228**	-.126**	.271**	.220**	.237**	1											
7. Meme and troll	-.091**	-.025	-.112**	-.049	-.056	-.182**	1										
8. COVID-19 concerning post	-.070*	-.004	-.268**	-.116**	-.073*	-.299**	-.060*	1									
9. Age^a^	.042	-.006	.005	-.046	-.040	-.059*	-.009	-.001	1								
10. Education^b^	.065*	.016	-.015	.078**	.037	-.056	.069*	.033	-.002	1							
11. Marital status	.055	-.014	-.038	-.006	-.053	-.073*	.011	-.007	.483**	.023	1						
12. Occupation	.050	-.018	-.063*	.005	-.019	-.040	-.004	.048	.367**	.017	.450**	1					
13. Smoking habit	.047	.008	-.018	-.050	-.074*	-.072*	-.013	.072*	-.078**	.003	-.069*	-.200**	1				
14. Stress^c^	.007	-.014	.014	-.007	-.019	-.013	.011	-.018	-.002	.007	.045	-.021	-.002	1			
15. Depression^c^	-.032	-.047	.056	-.013	-.005	.010	-.038	-.037	.040	-.045	.030	-.030	-.062*	.598**	1		
16. Anxiety^c^	-.044	-.037	-.059*	-.006	-.035	-.069*	-.015	.035	.089**	-.063*	.105**	.071*	-.115**	.530**	.554**	1	
17. Insomnia^c^	.020	-.046	-.011	.028	.006	-.015	.016	-.009	.021	-.007	.037	-.027	-.046	.456**	.490**	.481**	1

*significant at 0.05 level.

**significant at 0.01 level.

^a^age coded as 0 = under 30 years, 1 = above 30 years.

^b^education coded as 0 <graduate, 1 ≥ graduate.

^c^stress, depression, anxiety and insomnia are coded as per their level of condition.

From **Table 5**, which presents the seemingly unrelated multivariate logistic regression analyses, it is found that COVID-19-related facts and myths spread in social media, going to tour and no mask group photo were associated with anxiety but had no significant association with depression, stress and insomnia. Posting and sharing COVID-19-related memes and trolls had significant association with depression and anxiety. Being married was associated with all the mental health variables except depression and being non-smoker was associated with all the mental health variables except stress. Attending ceremonies, get together and COVID-19 concerning posts during this pandemic along with age, education and occupation had no significant association with any of the mental health variables.

**Table 5:** Seemingly unrelated multivariate logistic regression.

	Stress	Depression	Anxiety	Insomnia
	Odds Ratio[95% CI]	Odds Ratio[95% CI]	Odds Ratio[95% CI]	Odds Ratio[95% CI]
**Facts**	1.08[0.78, 1.49]	0.89[0.67, 1.18]	0.68** [0.50, 0.93]	1.11 [0.82, 1.51]
**Myths and misinformation**	0.89[0.52, 1.51]	0.71 [0.45, 1.12]	0.56**[0.33, 0.95]	0.76[0.45,1.26]
**Went to tour**	1.01 [0.67, 1.51]	1.01 [0.72, 1.42]	0.71* [0.48, 1.05]	0.84 [0.58, 1.23]
**Attended ceremony**	0.96[0.55,1.68]	0.82 [0.51, 1.33]	1.15 [0.68, 1.92]	1.13[0.68, 1.88]
**Get together**	0.80 [0.46, 1.37]	0.94 [0.60, 1.46]	0.89 [0.54, 1.46]	0.82 [0.50, 1.34]
**No mask group photo**	0.83[0.58, 1.20]	0.78 [0.57, 1.07]	0.63*** [0.44, 0.89]	1.02 [0.73, 1.42]
**Meme and troll**	0.99 [0.70, 1.39]	0.67*** [0.49, 0.90]	0.69** [0.49, 0.96]	1.09 [0.80, 1.50]
**COVID-19 concerning post**	0.85 [0.62, 1.16]	0.82 [0.63, 1.07]	1.01 [0.75, 1.36]	0.99 [0.74, 1.33]
**Age**	1.14[0.61,2.11]	1.17 [0.67, 2.05]	1.49 [0.84, 2.64]	1.47 [0.82, 2.63]
**Education**				
<Graduate	1.00(ref)			
≥ Graduate	1.13 [0.84, 1.52]	0.96 [0.75, 1.23]	0.84 [0.64, 1.10]	0.81 [0.62, 1.07]
**Marital status**				
Unmarried	1.00(ref)			
Married	1.44* [0.94, 2.20]	1.28 [0.88, 1.88]	1.60** [1.08, 2.39]	1.44* [0.96, 2.16]
**Occupation**				
Student	1.00(ref)			
Job	0.89 [0.60, 1.31]	0.81 [0.58, 1.13]	0.86 [0.60, 1.24]	0.77 [0.53, 1.11]
Business	0.71 [0.34, 1.50]	0.74 [0.40, 1.35]	1.24 [0.67, 2.31]	0.58 [0.29, 1.16]
**Smoking habit**				
Smoker	1.00(ref)			
Non-smoker	1.06 [0.75, 1.50]	0.69** [0.52, 0.93]	0.54*** [0.40, 0.74]	0.70** [0.51, 0.96]

## Discussion

Social media engagement in COVID-19 facts/myths and group photos without masks was associated with lower anxiety odds. Posting memes/trolls correlated with lower odds of anxiety and depression. Marital status linked to mental health factors, with married individuals having higher odds of anxiety, stress, and insomnia. Non-smokers had lower odds of depression, anxiety, and insomnia, except for stress. The second wave of COVID-19 in Bangladesh led to a nationwide seven-day restriction, impacting mental health of young social network users.

During the strict lockdown imposed amid the second wave of COVID-19 in Bangladesh, people increasingly engaged in social media, sharing a mix of factual COVID-19 information and false content, including posts with no-mask group photos, attending events, and ceremonies. In our study, 93.8% of participants were young, aligning with previous observations of daily social media users, which is in line a with previous related study [85]. The heightened reliance on the internet, particularly social media, was evident during the lockdown. Approximately 7.4% of shared posts propagated COVID-19 myths and misinformation, while recreational content, such as tour check-ins (16.8%), no-mask group photos (26.4%), and get-together programs (8.3%), constituted a significant portion. Notably, 21.7% of shared posts featured COVID-19-related trolls and memes, humorously addressing the pandemic. Our findings revealed that the most frequently shared posts were those related to COVID-19 concerns and facts, reflecting the diverse nature of social media content during these challenging times. Sharing falsified information related to COVID-19, facts related to COVID-19, careless behavior indicating posts comprising of no-mask group photos, get-together and ceremonies attending related posts were becoming their regular activities on social network Social network platform during lockdown pandemic situation. In our study we found that, 93.8% participants were young ages which is matched and corroborated to a previous observation of Rideout and Fox where they also reported that 93% of daily social media users were from the segment of young ages [85].

The current research reported that 7.9% of the misinformation was on COVID-19 vaccines. Vaccine conspiracy theories are actually not a new phenomenon. According to Wakefield et al. [86], receiving the MMR vaccine may result in autism. Following the release of this research paper, anti-vaccination movements gained popularity in the United States. Fortunately, investigative journalist Brian Deer was finally able to prove these results to be deceitful. The Lancet formally in 2010 withdrew this research article. Another myth about vaccine that has always been circulated is that vaccines are unsafe. An anecdote was surfaced and spread on social media after introduction of HPV vaccine that it induced chronic illness among adolescent female recipients [55,87–90] showed that most frequently shared fake information posts contents were related to vaccine conspiracy beliefs. The behavior of individuals was affected by health myths or conspiracy theories, which was explored by Jolly and Douglas [91]. The current research content analysis revealed that the contents of our research were consistent with previous research related to health issues. The current study also stated 15% myths that were comprised of false information about the origin of corona virus. Some previous seminal research studies reported similar type of myth where viral disease was supposed to be as a biological weapon against mankind. Moreover, another myth was spread about the Zika virus that it might jeopardize the population on earth [8,92].

Our research work also revealed that up to 14% myths were comprising of treatment rumors of COVID-19. During the outbreak of Ebola, treatment misinformation was replicated and spread on digital media. For instance, Fung et al. [93] and Pathak et al. [94] examined the role of some popular social platforms in spreading misinformation on treatments of Ebola. These studies also showed that much of this misinformation was very much influential and garnered much more popularity than facts. Personal opinion and, negative tones that are always reflected in the narratives of misinformation may induce emotional reactions and spread at a faster rate on social networks [95–100]. Several studies confirmed that hypothalamic pituitary adrenal (HPA) axis functioning is associated with such kind of self-reported prejudice. Any social threat is responsible for elevation of cortisol level that in-turn activates HPA axis [101–104]. Activation of HPA axis is an influential etiological cause of prejudicial attitudes [105].

During the pandemic of COVID-19, the substantial increase in social media based COVID-19’s myths and misinformation was a concerning matter for public health as it might induce disbelief towards health authorities, denial of government-imposed lockdown rules, obstacle to implement preventive measures to contain COVID-19 infection, vaccine hesitancy, and unnecessary delay in treatment. However, proper acquaintance with the phenomenon of dissemination of misinformation on social media during any pandemic, will play a vital role in elucidating the emergence and replication of future infodemics.

Our research survey demonstrated that about 32.5%, 8.4%, and 4.5% facts related posts were comprised of preventive measures of COVID-19, vaccines, and symptoms of COVID-19 respectively. Crook et al. [106] in their study on the 2014 Ebola outbreak stated about the immense capacity of social networks to combat health related misinformation through buttressing the public health regulatory authorities’ proclaimed facts on transmission, onset of symptoms, and preventive measures of disease. Another internet-based survey also revealed that about 12% individuals shared in a social media about their own health-related updates or followed health related updates of others [107]. Another study held back in 2010 by Scanfeld et al. reiterated that social media could be a hub to share information and advice related to health [108]. Two more studies explained how social media could combat misinformation during any health emergency [109,110]. Househ [111] in his study corroborated social media networks in fighting myths, as these tools can proclaim and support facts. Beneficial outcomes can be maximized by supporting and promoting the spread of facts related to the health and disease on social media, which in turn protect individuals from negative impacts of misinformation.

The overall situation during strict lockdown imposing days due to the advent of second wave in Bangladesh, most of the people were attracted to depend mostly on using internet especially social media platforms. People were busy in spending time in the social network platform to circulate different preventive and health concerning posts of COVID-19. In our research work, consequently same percentage representing posts (7.4%) were shared on social network platform indicating myths and misinformation spread regarding the COVID-19 infection and attending ceremonies during the pandemic situation. The findings of this study specifically divulged recreational posts on social network platform comprising of 16.8% tours’ check-in, 26.4% no-mask group photos, and 8.3% get together programs. The examined results of this study also indicated that 21.7% shared posts were related to COVID-19’s trolls and memes which in turn directly denigrated this pandemic situation on social network platform. After rigorous observation during the study period, we also found that two most frequently and successively shared posts on social network platform were COVID-19’s concerning posts and COVID-19’s facts respectively.

**Fig 2:**
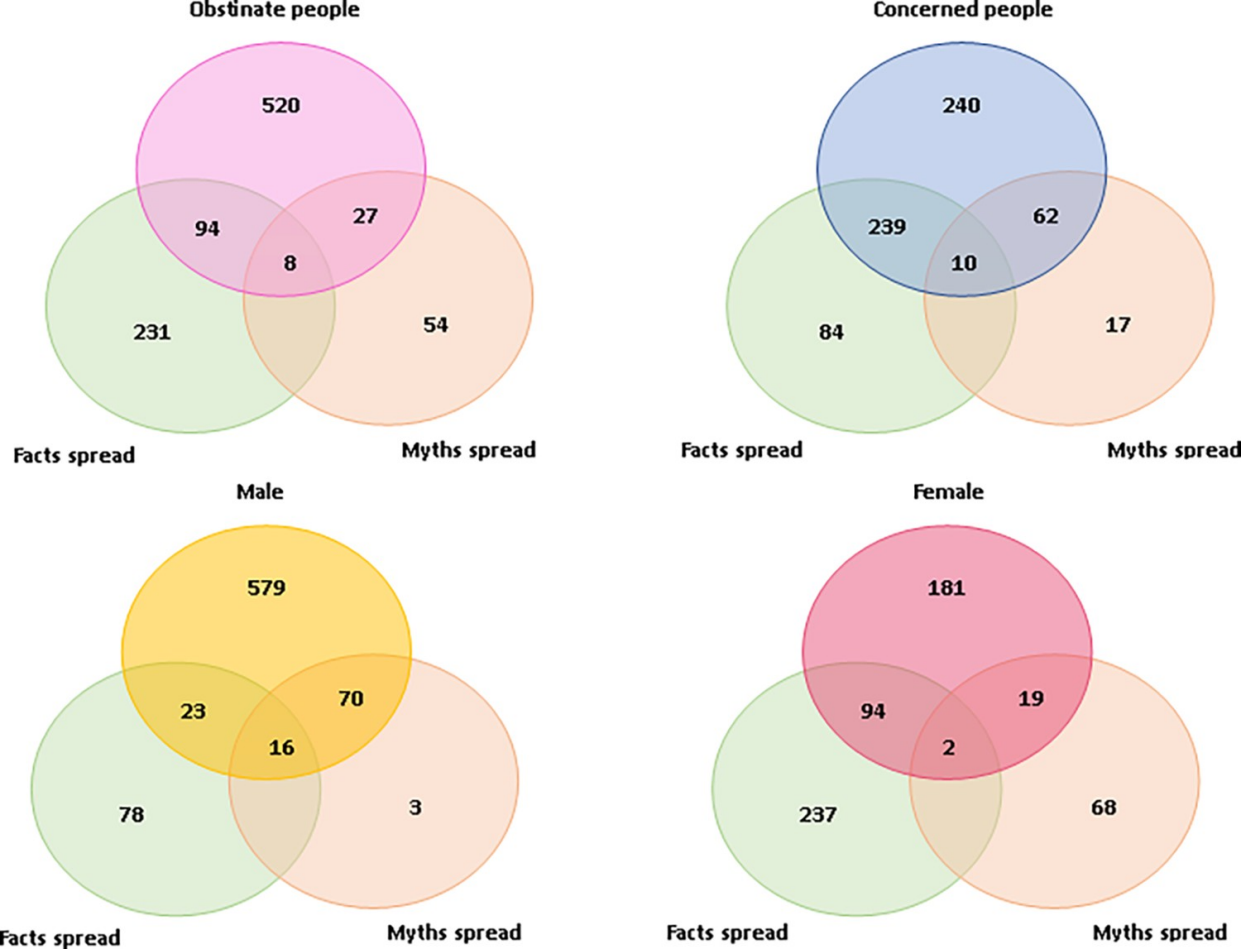
Venn diagram of spreading myths, facts or both on social media among obstinate & concerned people, male & female. Venn diagram illustrates the distribution of individuals who spread either facts or myths, or both, on social media platforms. The diagram differentiates between obstinate and concerned individuals within male and female populations, providing a numerical breakdown for each category.

Venn-diagrams are summarizing the actual sharing patterns of posts of myths and misinformation, facts among obstinate people, concerned people, males and females (**Fig 2**).

Among 649 obstinate individuals (54.08% of the study population), Venn diagrams illustrated that 1.23% simultaneously shared COVID-19 myths and facts on social media. In the remaining 45.92%, about 1.8% engaged in dual sharing. 4.16% of obstinate individuals shared COVID-19 myths, and 14.48% shared facts. Obstinate individuals, identified as jolly and unconcerned, showed lower preferences for sharing COVID-19 content compared to concerned individuals. For 551 concerned people (45.92% of the study population), 11.25% spread COVID-19 myths, while 43.38% shared facts. Pandemic-related fear and worry were linked to impaired information processing speed.

Concerned participants in our study exhibited a high prevalence of sharing both COVID-19 facts and myths, suggesting a slowed perception and response speed, possibly due to pandemic-related fear and worry. A Venn diagram highlighted gender differences, with 1.77% of males simultaneously sharing both COVID-19 myths and facts, while only 0.68% of females engaged in similar dual sharing on social media The high prevalence percentages of these posts of both COVID-19’s facts, and COVID-19’s myths and misinformation among concerned participants of this study clearly indicated their reduction in speed to perceive and respond for proper justification before circulating these on social network platform. Pandemic related worry and fear instigated them to share a plethora of COVID-19’s information they obtained from others.

Venn-diagram also revealed a clear distinction between males and females inclined to share COVID-19’s facts, COVID-19’s myths and misinformation on social network platform. We found relatively higher likelihoods of simultaneously sharing posts of both COVID-19’s myths and misinformation, and COVID-19’s facts among 1.77% males on social network. Although, only 0.68% females were involved in simultaneously circulating both COVID-19’s myths and misinformation, and COVID-19’s facts on social network platform.

Among social media users posting about COVID-19, 2.8% had severe insomnia, aligning with a larger population study in 2021 [112]. Subthreshold and moderate insomnia were found in 19.1% and 8.2%, respectively, totaling 30.1%, consistent with the reported rates of a study [113]. Depressive symptoms were prevalent at 43.9%, matching findings from a 2020 study in Bangladesh [114]. Stress levels (23.8%) were comparable to national rates (25.38%) but lower than the reported overall stress level (59.7%) in Bangladesh during the pandemic [115].

The current findings indicate that 30.9% of respondents suffered mild to extremely severe anxiety symptoms. Several cross-sectional studies have reported 37%, 39.8% and 46% prevalence rates of anxiety symptoms among general population in Bangladesh [26,32,115]. The prevalence of stress level was much lower in the present study than the reported studies in Bangladesh during COVID-19 outbreak in 2020. Several previously performed prominent studies stated age as the significant influencer of mental health of people [26,30,115–119]. Consequently, in our present study we have seen most of the participants were within an age range spanning approximately 21–29, which is in line with a study performed by Islam et al. in 2020 where they confirmed that participants aged between 21 to 30 years were at a lower risk of developing anxiety symptoms [117].

This research indicates lower stress, depression, and anxiety rates compared to other studies in Bangladesh, suggesting a balanced lifestyle and quality time with family and friends amid the ongoing pandemic. Adapting to the uncertainty of the situation may mitigate the risk of severe mental health problems. Surprisingly, 34.4% of concerned respondents experienced anxiety, higher than the 28% of obstinate respondents. Stress and insomnia showed similar levels among obstinate and concerned individuals. However, 45.3% of concerned participants experienced depression, slightly higher than the overall and obstinate participants’ scores (43.9% and 42.8%, respectively). COVID-19 not only poses a global health threat but also adversely affects mental health and well-being.

Another study reported that fear of getting contracted an infection resulted in the prevalence of both anxiety and depression symptoms [120]. Consequently, a review study reported that depression and anxiety symptoms in different countries may vary from 14.6% to 48.3% and from 33% to 50.9% respectively [121]. Anxiety levels of concerned individuals clearly fall within the indicated range of Xiong et al. [121].

Dissemination of COVID-19 misinformation on social media poses a significant public health threat, with potential life-endangering consequences. BBC.com reported 800 global deaths in August 2020 due to coronavirus-related misinformation [122]. Our study observed minimal vaccine misinformation sharing. However, 10.1% shared posts promoting alcohol consumption to prevent transmission, reflecting alarming health risks. Reports from Al Jazeera highlighted deaths in Iran from toxic methanol consumption based on COVID-19 misinformation [123]. The 3rd highest shared posts focused on unconventional treatments, contributing to health hazards. The 2nd highest shared posts fueled racial hatred by blaming China for intentional COVID-19 spread [124]. The most shared myths denigrated the virus, ironically claiming it only affected the elite class. Popular social media platforms, notably Facebook, play a significant role in amplifying misinformation, emphasizing the urgent need for mitigation strategies [125].

Our study found that COVID-19 facts shared on social media focused on six topics: preventive measures, vaccine updates, virus origin, pandemic scenarios, symptoms, and educational institution openings in Bangladesh. A significant number of respondents emphasized preventive measures, particularly promoting mask-wearing, social distancing, and frequent handwashing. This awareness aimed to combat COVID-19 with limited resources in Bangladesh, emphasizing the importance of avoiding mass gatherings. The second most shared facts were related to vaccines, with users disseminating information on safety, efficacy, and WHO approval dates. Notably, news in August 2021 reported two crore COVID-19 vaccination registrations in Bangladesh through the Surokkha app [126]. This highlights the inefficacy of spreading vaccine myths on social media.

Our study found vaccine facts were four times more prevalent than vaccine misinformation. The third most shared facts were related to the origin of COVID-19, with users prioritizing WHO’s news on SARS-CoV-2’s animal origin, dismissing claims of manipulation or lab construction. Suspected COVID-19 symptoms prompt testing, enabling early isolation and treatment, reducing severe outcomes. Bangladesh increased test centers due to rising demand for confirmation of symptoms. Few social media posts discussed the reopening of educational institutions, reflecting concerns of parents and students. Females more actively shared COVID-19 facts on social platforms than males. Gender-based insomnia risk was higher among females, aligning with other Bangladeshi studies during the pandemic [112,127–129].

Another meta-analysis of observational Studies performed after reviewing 13 articles back in 2020 showed higher numbers of females was significantly remained insomniacs compared with males [130]. More studies also found similar results that females had been suffering from insomnia more frequently than males [131–133]. 37.9% of females in our study had insomnia, with much lower rates (27.6%) among males. Various factors contribute to higher insomnia in females. Additionally, 7.4% and 6.1% of females experienced extremely severe depression and anxiety, surpassing male rates. Multiple studies also confirm females’ elevated risk for depression and anxiety symptoms [134–136]. These psychiatric problems in females also might contribute to increase the risk of developing insomnia. According to our study, social network users suffering from insomnia also had significant association with stress, depression and anxiety.

In our study, 31.8% of females shared facts on social platforms, while 26.4% of males spread myths and misinformation. Females demonstrated greater accuracy in sharing COVID-19 information, resulting in fewer instances of misinformation compared to male users in Bangladesh. The Centre for Countering Digital Hate found that only 12.5% of identified misinformation-related posts were acted upon by Facebook, Instagram, YouTube, and Twitter [137]. These scenarios revealed how these social media companies are struggling to keep the spreading of these myths and misinformation in check. Almenar E and colleagues in 2021 in their study entitled Gender differences in tackling fake news: different degrees of concern, but same problems reported that females were more concerned than males about detrimental consequences of misinformation [138].

At a 0.001 significance level, the no-mask group shared COVID-19 myths, tours’ check-ins, and attended events on social media. Another subset of the no-mask group shared COVID-19 facts. Those who posted tour check-ins were inclined to share memes. Users with anxiety symptoms significantly shared COVID-19 concerns and had smoking habits. A WHO study in 2020 found that news followers of COVID-19 experienced the most anxiety [139]. In our study, social network users with anxiety symptoms significantly had both stress and depression symptoms. Some recent studies also had stated similar results describing COVID-19 induced anxiety, depression and stress [140–142].

### Implications

We anticipate that this study’s findings will contribute to our understanding of how social media-based facts, myths, and misinformation, as well as obstinate posts that violate preventive measures and worrisome posts, can impact people’s stress, anxiety, depression, and sleep quality during a pandemic and potentially undermine all future attempts to prevent infection. The results of this study can be an example for any health concerned groups for preparing and responding properly in any health-related emergency to properly handle misinformation, facts related posts on social media platforms and maintain a sound psychological conditions among its users [143].

The negative impact of misinformation, obstinate and concerning posts on insomnia, depression, stress, and anxiety of social network users can ultimately influence the dissemination of such activities in several social platforms. However, disease management committee may establish strategies to provide timely and scientific evidence-based information to fight against the exposure of disease related myths and misinformation on social networks during any health crisis moment. In addition to the theoretical implications, it is our believe that the content observation and analysis technique of our study showed how to identify and categorize facts and myths on social media platforms during any pandemic situation. This technique could allow disease and health management authorities to assess the posts indicating disease and health related misinformation and myths, and to execute right actions to prevent its further proliferation on social networks.

One of the possible and fruitful actions to reduce myths and misinformation sharing along with its negative psychological impacts is to regulate social network users who are at risk to share disease or health related misinformation [144]. For instance, Ozturk et al. in their study reported that warning signs generated against any posts containing misinformation likely to decrease its further dissemination on social media [145]. Bode and Vraga (2018) also reiterated on the importance of algorithmic correction in social network to effectively reduce the spread of misinformation [146]. We believe that health and disease awareness campaigns to raise awareness on checking the scientific validity of any posts while sharing or believing any pandemic related information, could minimize the prevalence percentages of myths and misinformation related to diseases as well as negative psychological effects among social network users. Besides all these promising interventions, the reduction of both duration and exposure frequency of social network, assimilation of scientific evidence based COVID-19 like health and disease related information are some beneficial advice for individuals who spread information during COVID-19 like pandemic situation.

### Limitations

The main limitation of this research work is to involve comparatively smaller numbers of female respondents which was almost one third of male respondents. This poor participation number of females might have been due to their privacy concerns in social networks. In addition, in-person or face-to-face interviewing session was not possible with the onset of the second wave of COVID-19 in Bangladesh. Furthermore, this study did not compare the psychological state with the post-spreading conditions following the end of the COVID-19 pandemic. Unaddressed potential confounders add another layer of limitation, recognizing the need for cautious interpretation of the study’s outcomes.

### Future scopes

In future research, we will endeavor to expand our research beyond observing contents of posts shared on social networks and consider different reactions in the posts indicating misinformation or distinctive comments on it. For instance, reactions and comments will be suitably examined to determine whether they endorse, oppose, or remain neutral towards the posts that contain misinformation. Furthermore, our goal is to understand how users in general, healthcare professionals, and patients respond to misinformation about health and illness on social media. Another research direction includes assessing the adaptation process of all the proposed intervention strategies to successfully prevent the spreading of health-related misinformation on social networks during any emergency crisis. Future research should address the gender disparity in participation, exploring ways to enhance female engagement on social networks. Conducting in-person interviews and considering potential confounders will strengthen the study design. Further investigations could delve into the specific content that influences mental health outcomes, aiding in the development of targeted interventions to mitigate stress, anxiety, depression, and insomnia during pandemics.

## Conclusion

To the best of our knowledge, this is the first study where we extensively scrutinize and assess not only the mental health status of selected active social network users but also their disseminated COVID-19’s myths and misinformation, COVID-19’s facts, concerning and obstinate posts on social media during the second wave of COVID-19 in Bangladesh. Our study focused mainly on pointing out the most frequently shared contents of myths and misinformation, facts about COVID-19. Female participants reported a high awareness about COVID-19’s myths and misinformation and divulging themselves as highly capable individuals while differentiating COVID-19’s myths and misinformation against COVID-19’s facts and circulating highest percentages of accurate COVID-19’s information. Surprisingly, despite this, a substantial proportion of female respondents reported to demonstrate relatively higher prevalence rates of stress, depression, anxiety and insomnia symptoms in comparison to male respondents. It is puzzling that both concerned and obstinate individuals exhibited similar prevalence rates in terms of stress, insomnia but exhibited slight difference in terms of depression level and maintained a disparity in terms of anxiety level. Sharing percentages of COVID-19’s myths and misinformation over COVID-19’s facts by obstinate and concerned respondents clearly highlighting that concerned respondents were inclined to share more frequently both COVID-19’s myths and misinformation and COVID-19’s facts simultaneously. The spread of myths and misinformation on social media undermines any efforts to curb COVID-19 infection by circulating COVID-19’s preventive measures, facts and update of COVID-19’s immunization through vaccination, new variant’s sign and symptoms. Our study findings underscore the importance of using fact checking facilities to maintain and ensure the credibility of any related health information, which is sufficient enough to nearly prevent individuals from sharing myths and misinformation on social media platform. It therefore be said that instantaneous intervention can keep the spread of myths and misinformation about COVID-19 in check and promote the circulation of COVID-19’s facts on social media.

## Supporting information

S1 File This file contains the English version of the study questionnaire. The questionnaire comprises with three sections: Socio-demographic data, Personal life preferences during COVID-19, and Psychometric indices.
(DOCX)

S2 File This file contains the Bangla version (Native language) of the study questionnaire. The questionnaire comprises with three sections: Socio-demographic data, Personal life preferences during COVID-19, and Psychometric indices.
(DOCX)

S1 Data The dataset is coded unanimously with SPSS and all personal information was excluded during coding the data. This dataset was used for statistical analysis.
(SAV)

## Abbreviations

COVID-19: Corona Virus Disease of 2019
H1N1: hemagglutinin (H) and neuraminidase (N)
ISI: Insomnia Severity Index
DASS21: Depression, Anxiety and Stress Scale
SD: Standard Deviation
WHO: World Health Organization
SARS-CoV-2: Severe acute respiratory syndrome coronavirus 2
ICU: Intensive Care Unit
CCDH: Centre for Countering Digital Hate
