# Supplementary material for: COVID-19’s myths, facts, concerning and obstinate posts on social network, and the mental health status of social network users in Bangladesh
Source: PLOS Ment Health. 2024 Jun 24;1(1):e0000014. doi: 10.1371/journal.pmen.0000014 (PMC12798192; doi:10.1371/journal.pmen.0000014)
Supplement: S1 File — The questionnaire comprises with three sections: Socio-demographic data, Personal life preferences during COVID-19, and Psychometric indices. (DOCX) [file pmen.0000014.s001.docx]

**Part - 1: Socio-demographic data**

Name: ___________________________________________________________

Gender:

- Male
- Female
- Other

Age: ______________________ years

Marital Status:

- Married
- Unmarried
- Divorced
- Widow/ Widower

Educational Qualification:

- Never went to school
- Primary
- Secondary (SSC)
- Higher Secondary (HSC)
- Honors
- Masters
- PhD

Present Address (Only District): ___________________________________________

Occupation:

- Student
- Job
- Business
- Housewife
- Unemployed

Smoking habit:

- Smoker
- Non-smoker

**Part - 2: Personal life preference during COVID-19**

Total number of tours you went during this COVID-19 pandemic -

- Never
- 1
- 2
- 3
- 4
- ≥ 5

Total number of ceremonies/ anniversaries/ family programs you attended during this COVID-19 pandemic -

- Never
- 1
- 2
- 3
- 4
- ≥ 5

Total numbers of Get-together programs with friends or other members you attended during this COVID-19 pandemic -

- Never
- 1
- 2
- 3
- 4
- ≥ 5

**Part - 3: Psychometric indices**

**DASS21**

1. I found it difficult to relax

- Never
- Sometimes
- Often
- Almost always

2. I tended to over-react to situations

- Never
- Sometimes
- Often
- Almost always

3. I felt that I was using a lot of nervous energy

- Never
- Sometimes
- Often
- Almost always

4. I found myself getting agitated

- Never
- Sometimes
- Often
- Almost always

5. I found it hard to wind down

- Never
- Sometimes
- Often
- Almost always

6. I was intolerant of anything that kept me from getting on with what I was

Doing

- Never
- Sometimes
- Often
- Almost always

7. I felt that I was rather touchy

- Never
- Sometimes
- Often
- Almost always

8. I couldn’t seem to experience any positive feeling at all

- Never
- Sometimes
- Often
- Almost always

9. I found it difficult to work up the initiative to do things

- Never
- Sometimes
- Often
- Almost always

10. I felt that I had nothing to look forward to

- Never
- Sometimes
- Often
- Almost always

11. I felt down-hearted and blue

- Never
- Sometimes
- Often
- Almost always

12. I was unable to become enthusiastic about anything

- Never
- Sometimes
- Often
- Almost always

13. I felt I wasn’t worth much as a person

- Never
- Sometimes
- Often
- Almost always

14. I felt that life was meaningless

- Never
- Sometimes
- Often
- Almost always

15. I was aware of dryness of my mouth

- Never
- Sometimes
- Often
- Almost always

16. I experienced breathing difficulty (eg, excessively rapid breathing, breathlessness in the absence of physical exertion)

- Never
- Sometimes
- Often
- Almost always

17. I experienced trembling (eg, in the hands)

- Never
- Sometimes
- Often
- Almost always

18. felt I was close to panic

- Never
- Sometimes
- Often
- Almost always

19. I was worried about situations in which I might panic and make a fool of Myself

- Never
- Sometimes
- Often
- Almost always

20. I was aware of the action of my heart in the absence of physicalexertion (eg,

sense of heart rate increase, heart missing a beat)

- Never
- Sometimes
- Often
- Almost always

21. I felt scared without any good reason

- Never
- Sometimes
- Often
- Almost always

**ISI**

1. Difficulty falling asleep

- None
- Mild
- Moderate
- Severe
- Very severe

2. Difficulty staying asleep

- None
- Mild
- Moderate
- Severe
- Very severe

3. Problems waking up too early

- None
- Mild
- Moderate
- Severe
- Very severe

4. How SATISFIED/DISSATISFIED are you with your last 2 weeks sleep pattern?

- Very satisfied
- Satisfied
- Moderately satisfied
- Dissatisfied
- Very Dissatisfied

5. To what extent do you consider your sleep problem to INTERFERE with your daily functioning (e.g. daytime fatigue, mood, ability to function at work/daily chores, concentration, memory, mood, etc.) last 2 weeks?

- Not at all interfering
- A little
- Somewhat
- Much
- Very much interfering

6. How NOTICEABLE to others do you think your sleep problem is in terms of impairing the quality of your life?

- Not at all Noticeable
- A little
- Somewhat
- Much
- Very much noticeable

7. How WORRIED/DISTRESSED are you about your last two weeks sleep problem?

- Not at all worried
- A little
- Somewhat
- Much
- Very much worried
