# Supplementary material for: COVID-19’s myths, facts, concerning and obstinate posts on social network, and the mental health status of social network users in Bangladesh
Source: PLOS Ment Health. 2024 Jun 24;1(1):e0000014. doi: 10.1371/journal.pmen.0000014 (PMC12798192; doi:10.1371/journal.pmen.0000014)
Supplement: S2 File — The questionnaire comprises with three sections: Socio-demographic data, Personal life preferences during COVID-19, and Psychometric indices. (DOCX) [file pmen.0000014.s002.docx]

**১ম অংশ: আর্থ-সামাজিক তথ্য**

নাম: ___________________________________________________________

লিঙ্গ:

- পুরুষ
- নারী
- অন্যান্য

বয়স: ______________________ বছর

বৈবাহিক অবস্থা:

- বিবাহিত
- অবিবাহিত
- তালাকপ্রাপ্ত
- বিধবা/ বিপত্নীক

শিক্ষাগত যোগ্যতা:

- কখনো স্কুলে যাই নি
- প্রাথমিক
- মাধ্যমিক (এসএসসি)
- উচ্চ মাধ্যমিক (এইচএসসি)
- স্নাতক (সম্মান)
- স্নাতকোত্তর
- পিএইচডি

বর্তমান ঠিকানা (শুধুমাত্র জেলা): ___________________________________________

পেশা:

- শিক্ষার্থী
- চাকুরি
- ব্যবসা
- গৃহিণী
- বেকার

ধূমপানের অভ্যাস:

- ধূমপায়ী
- অধূমপায়ী

**২য় অংশ: করোনাকালিন সময়ের ব্যক্তিজীবন সম্পর্কিত তথ্য**

করোনা পরিস্থিতিতে মোট কতবার ভ্রমণে গিয়েছিলেন?

- একবারও না
- ১ বার
- ২ বার
- ৩ বার
- ৪ বার
- ৫ বা তার বেশি বার

করোনা পরিস্থিতিতে মোট কতগুলো অনুষ্ঠানে অংশগ্রহণ করেছেন?

- একটিও না
- ১ টি
- ২ টি
- ৩ টি
- ৪ টি
- ৫ টি বা তার বেশি

করোনা মহামারি অবস্থায় বন্ধু বা অন্যদের সাথে মোট কতবার একত্রিত হয়েছিলেন?

- একবারও না
- ১ বার
- ২ বার
- ৩ বার
- ৪ বার
- ৫ বা তার বেশি বার

**৩য় অংশ: মানসিক অবস্থা সূচক**

**DASS21**

১। করোনাকালিন এই উৎকন্ঠার মধ্যে নিজেকে আগের মতো শান্ত ও স্বাভাবিক অবস্থায় ফিরে আনা আপনার জন্য কঠিন কিছু কি?

- কখনই না
- মাঝে মাঝে
- প্রায়ই
- প্রায় সবসময়

২। করোনা মহামারিতে চাপের কারণে আপনার মধ্যে বিভিন্ন পরিস্থিতিতে অতিরিক্ত প্রতিক্রিয়া করার প্রবনতা আছে কি?

- কখনই না
- মাঝে মাঝে
- প্রায়ই
- প্রায় সবসময়

৩। করোনা মহামারিতে আপনার মনে হয় যে আপনি খুব বেশী স্নায়ু চাপে ভুগছেন?

- কখনই না
- মাঝে মাঝে
- প্রায়ই
- প্রায় সবসময়

৪। করোনার এই পরিস্থিতিতে আপনি উৎকন্ঠিত ও অস্থির হয়ে যাচ্ছেন কি?

- কখনই না
- মাঝে মাঝে
- প্রায়ই
- প্রায় সবসময়

৫। করোনার এখানকার পরিস্থিতিতে আপনি স্নায়বিক বা মানসিক চাপ বোধ করেন কি?

- কখনই না
- মাঝে মাঝে
- প্রায়ই
- প্রায় সবসময়

৬। করোনার এই পরিস্থিতিতে আপনার অনেক কাজ আটকে থাকায় এই সময়টাকে কি আপনার কাছে অসহ্য লাগছে?

- কখনই না
- মাঝে মাঝে
- প্রায়ই
- প্রায় সবসময়

৭। আপনার কি নিজেকে একটু বেশি আবেগ প্রবন অভিমানী বলে মনে হয়?

- কখনই না
- মাঝে মাঝে
- প্রায়ই
- প্রায় সবসময়

৮। করোনা মহামারিতে ইতিবাচক কোন অনুভূতি বা চিন্তা আপনার মধ্যে সহজে আনতে পারেন না এমন হয়েছে কি?

- কখনই না
- মাঝে মাঝে
- প্রায়ই
- প্রায় সবসময়

৯। করোনা মহামারিতে মনমরা থাকায় নিজে থেকে উদ্যোগী হয়ে যে কোন কাজ শুরু করা কে আপনি কঠিন কিছু মনে করেন কি?

- কখনই না
- মাঝে মাঝে
- প্রায়ই
- প্রায় সবসময়

১০। করোনার এই পরিস্থিতিতে ভবিষ্যতে আপনি ভাল কিছুরই আশা হারিয়ে ফেলছেন কি?

- কখনই না
- মাঝে মাঝে
- প্রায়ই
- প্রায় সবসময়

১১। করোনার এখনকার পরিস্থিতিতে আপনি মনমরা এবং বিষন্ন অনুভব করেন কি?

- কখনই না
- মাঝে মাঝে
- প্রায়ই
- প্রায় সবসময়

১২। করোনার এই পরিস্থিতিতে আপনি কি কোন কিছুতেই বেশী আগ্রহী বা উৎসাহিত হতে পারছেন না?

- কখনই না
- মাঝে মাঝে
- প্রায়ই
- প্রায় সবসময়

১৩। করোনার এই পরিস্থিতিতে আপনার নিজেকে কখনো মূল্যহীন বলে মনে হয়েছে কি?

- কখনই না
- মাঝে মাঝে
- প্রায়ই
- প্রায় সবসময়

১৪। করোনার এই পরিস্থিতিতে জীবনটাকে কি আপনার এখন অর্থহীন বলে মনে হয়?

- কখনই না
- মাঝে মাঝে
- প্রায়ই
- প্রায় সবসময়

১৫। করোনার এই সময়ে আপনার গলা কি কখনো আবেগ উৎকন্ঠায় শুকিয়ে আসে? *

- কখনই না
- মাঝে মাঝে
- প্রায়ই
- প্রায় সবসময়

১৬। করোনার এই সময়ে শারীরিক পরিশ্রম ছাড়াই কখনো কি আপনার নিঃশ্বাস প্রায় বন্ধ হয়ে এসেছে, এবং অতিদ্রুত শ্বাসপ্রশ্বাস নিতে হয়েছে?

- কখনই না
- মাঝে মাঝে
- প্রায়ই
- প্রায় সবসময়

১৭। করনায় উদ্বিগ্ন হয়ে আপনার শরীর ও হাত কাঁপার অভিজ্ঞতা হয়েছে কি? *

- কখনই না
- মাঝে মাঝে
- প্রায়ই
- প্রায় সবসময়

১৮। করোনায় দুশ্চিন্তাগ্রস্ত হয়ে আপনি তীব্রভাবে আতঙ্কিত হয়ে পরেছেন কি?

- কখনই না
- মাঝে মাঝে
- প্রায়ই
- প্রায় সবসময়

১৯। করোনার এই পরিস্থিতির কারণে আপনার কি মনে হয় আপনি হঠাৎই আতঙ্কগ্রস্ত হয়ে যাচ্ছেন?

- কখনই না
- মাঝে মাঝে
- প্রায়ই
- প্রায় সবসময়

২০। করোনার এই পরিস্থিতিতে আপনি কোন শারীরিক পরিশ্রম না করা সত্ত্বেও “আপনার হৃদপিন্ডের স্পন্দন স্বাভাবিক এর চেয়ে বেড়ে যায় বা বুক ধরফর করতে থাকে” এরকমটা কখন হয়েছে কি?

- কখনই না
- মাঝে মাঝে
- প্রায়ই
- প্রায় সবসময়

২১। করোনার এই পরিস্থিতিতে যথাযথ কোনো কারন ছাড়াই আপনি ভীত-সন্ত্রস্ত হয়ে যান কি?

- কখনই না
- মাঝে মাঝে
- প্রায়ই
- প্রায় সবসময়

**ISI**

১। করোনার কারণে দেখা যাচ্ছে আগের মতোন বিছানায় শোবার সাথে সাথে ঘুম আসছে না, দেরি হচ্ছে।

- মোটেই না
- কিছুটা
- মোটামুটি
- গুরুতর
- খুবই গুরুতর

২। করোনার এই সময়টাতে দেখা যাচ্ছে আমি আগের মতোন খুব বেশি সময় পর্যন্ত ঘুমিয়ে থাকতে পারছি না।

- মোটেই না
- কিছুটা
- মোটামুটি
- গুরুতর
- খুবই গুরুতর

৩। করোনার এই সময়ে বিনা কারণেই খুব দ্রুতই ঘুম ভেঙে যাচ্ছে।

- মোটেই না
- কিছুটা
- মোটামুটি
- গুরুতর
- খুবই গুরুতর

৪। করোনার এই পরিস্থিতিতে আপনার গত ২ সপ্তাহের ঘুমের অবস্থা নিয়ে আপনি কি সন্তুষ্ট?

- খুবই সন্তুষ্ট
- সন্তুষ্ট
- মোটামুটি সন্তুষ্ট
- অসন্তুষ্ট
- খুবই অসন্তুষ্ট

৫। করোনা এর এই মহামারি পরিস্থিতিতে আপনার ঘুমের সমস্যা কি আপনার দৈনন্দিন কাজে কোনো ব্যাঘাত ঘটায়?

- মোটেও ব্যঘাত ঘটায় না
- সামান্য ব্যঘাত ঘটায়
- মোটামুটি ব্যঘাত ঘটায়
- বেশি ব্যঘাত ঘটায়
- অনেক বেশি ব্যঘাত ঘটায়

৬। “অনিদ্রার কারণে আপনার দৈনন্দিন কাজের যে ক্ষতি হচ্ছে”এই ব্যাপারটা কি আপনার পরিবারের অন্য সকল সদস্যের কাছে লক্ষণীয়?

- মোটেও লক্ষ্যণীয় না
- সামান্য লক্ষ্যণীয়
- মোটামুটি লক্ষ্যণীয়
- বেশি লক্ষ্যণীয়
- অনেক বেশি লক্ষ্যণীয়

৭। করোনা এর এই পরিস্থিতিতে আপনি কতটুকু চিন্তিত/উদ্বিগ্ন আপনার ঘুমের সমস্যার জন্য?

- মোটেও চিন্তিত না
- সামান্য চিন্তিত
- মোটামুটি চিন্তিত
- বেশি চিন্তিত
- অনেক বেশি চিন্তিত
